# A nationwide survey of hereditary angioedema due to C1 inhibitor deficiency in Italy

**DOI:** 10.1186/s13023-015-0233-x

**Published:** 2015-02-06

**Authors:** Andrea Zanichelli, Francesco Arcoleo, Maria Pina Barca, Paolo Borrelli, Maria Bova, Mauro Cancian, Marco Cicardi, Enrico Cillari, Caterina De Carolis, Tiziana De Pasquale, Isabella Del Corso, Paola Cesinaro Di Rocco, Maria Domenica Guarino, Ilaria Massaro, Paola Minale, Vincenzo Montinaro, Sergio Neri, Roberto Perricone, Stefano Pucci, Paolina Quattrocchi, Oliviero Rossi, Massimo Triggiani, Giuseppina Zanierato, Alessandra Zoli

**Affiliations:** Dipartimento di Scienze Biomediche e Cliniche “Luigi Sacco”, Università degli Studi di Milano, Ospedale Luigi Sacco, Milano, Italy; U.O.C. Patologia Clinica, Ospedali Riuniti Villa Sofia-Cervello, Palermo, Italy; Struttura complessa di Medicina Interna e Allergologia e Immunologia Clinica, AUO Cagliari, Cagliari, Italy; Struttura Complessa Medicina Interna, Ambulatorio di Allergologia, Ospedale U. Parini, Aosta, Italy; Centro Interdipartimentale di Ricerca in Scienze Immunologiche di Base e Cliniche, A.O.U. Federico II, Naples, Italy; Dipartimento di Medicina, Università degli Studi di Padova, Padova, Italy; UOC Ginecologia ed Ostetricia II Azienda Ospedaliera San Giovanni-Addolorata Roma, Rome, Italy; Unità Operativa di Allergologia, Presidio ospedaliero di Civitanova Marche, Civitanova Marche, Italy; UO Immuno-Allergologia, Dipartimento di Medicina Interna, Azienda Ospedaliera Universitaria Pisana (AOUP), Pisa, Italy; U.O.D.S. di Allergologia Clinica AUSL, Pescara, Italy; UOC Reumatologia, Policlinico Tor Vergata, Roma, Italy; Centro di Ricerca, Trasferimento ed Alta Formazione Denothe, Università degli Studi di Firenze, Firenze, Italy; IRCCS San Martino, Dipartimento di Medicina Interna UOC Allergologia, Genova, Italy; Unità Operativa Nefrologia Universitaria Azienda Ospedaliero-Universitaria “Consorziale Policlinico” Bari, Bari, Italy; Dipartimento di Scienze mediche e pediatriche, Medicina Interna “A Francaviglia”, Policlinico universitario di Catania, Catania, Italy; U.O.C. di Allergologia e Immunologia Clinica, Dipartimento di Medicine Specialistiche, Policlinico Universitario, Messina, Italy; SOD Immunoallergologia AOU Careggi, Florence, Italy; Dipartimento di Medicina e Chirurgia, Università degli Studi di Salerno, Salerno, Italy; Struttura Semplice Allergologia, Azienda Sanitaria Locale di Biella, Biella, Italy; Servizio di Immunologia Clinica e Tipizzazione Tessutale- Ospedali Riuniti di Ancona, Ancona, Italy

**Keywords:** Hereditary angioedema, C1 inhibitor, C4

## Abstract

**Introduction:**

Hereditary angioedema due to C1-inhibitor deficiency (C1-INH-HAE type I) or dysfunction (C1-INH-HAE type II) is a rare disease characterized by recurrent episodes of edema with an estimated frequency of 1:50,000 in the global population without racial or gender differences. In this study we present the results of a nationwide survey of C1-INH-HAE patients referring to 17 Italian centers, the Italian network for C1-INH-HAE, ITACA.

**Methods:**

Italian patients diagnosed with C1-INH-HAE from 1973 to 2013 were included in the study. Diagnosis of C1-INH-HAE was based on family and/or personal history of recurrent angioedema without urticaria and on antigenic and/or functional C1-INH deficiency.

**Results:**

983 patients (53% female) from 376 unrelated families were included in this survey. Since 1973, 63 (6%) patients diagnosed with C1-INH-HAE died and data from 3 patients were missing when analysis was performed. Accordingly, the minimum prevalence of HAE in Italy in 2013 is 920:59,394,000 inhabitants, equivalent to 1:64,935. Compared to the general population, patients are less represented in the early and late decades of life: men start reducing after the 5^th^ decade and women after the 6^th^. Median age of patients is 45 (IQ 28-57), median age at diagnosis is 26 years (IQ 13-41). C1-INH-HAE type 1 are 87%, with median age at diagnosis of 25 (13-40); type 2 are 13% with median age at diagnosis of 31 (IQ 16-49). Functional C1INH is ≤50% in 99% of patients. Antigen C1INH is ≤50% in 99% of type 1. C4 is ≤50% in 96% of patients. The chance of having C1-INH-HAE with C4 plasma levels >50% is < 0.05.

**Conclusion:**

This nationwide survey of C1-INH-HAE provides for Italy a prevalence of 1:64,935. C1-INH-HAE patients listed in our database have a shorter life expectancy than the general population. An increased awareness of the disease is needed to reduce this discrepancy. Measurement of C4 antigen can exclude diagnosis of C1-INH-HAE with an accuracy > 95%. This parameter should be therefore considered for initial screening in differential diagnosis of angioedema.

## Background

Hereditary angioedema (HAE) due to C1 Inhibitor (C1-INH) deficiency (C1-INH-HAE) is a rare autosomal dominant disease due to reduced C1-INH plasma levels (C1-INH-HAE type I) or to the presence of a dysfunctional C1-INH (C1-INH-HAE type II) [1]. C1-INH-HAE type I is estimated to occur in approximately 85% of patients, type II occurs in the remaining 15%. C4 is reduced in both C1-INH-HAE type I and II while C3 is normal [2]. The disease is caused by mutation in C1-INH gene (*SERPING1*). *De novo* mutants, i.e. the subjects whose parents have a normal C1-INH, can be identified in approximately 25% of the families [3]. The deficiency of C1-INH results in uncontrolled activation of the contact system and release of bradykinin, the mediator of increased vascular permeability and angioedema manifestations [4,5]. C1-INH-HAE manifests with recurrent episodes of edema of the skin, gastrointestinal tract and upper airway. The disease is disabling and laryngeal edema can lead to asphyxiation and death if left untreated [2].

Data on the prevalence of C1-INH-HAE are sparse. The estimated frequency worldwide reported in literature varies from 1 every 10,000 to 1 every 150,000 persons without racial or gender differences [6-8]. A nationwide survey in Denmark, based on 76 HAE patients, reported a minimum prevalence of 1:70,922 inhabitants [9]. Previous Norwegian and Spanish studies based on 67 and 444 patients respectively found a minimal prevalence of 1:66,225 and 1:91,743 inhabitants [10,11]. Two more recent surveys in Sweden and in Slovenia based respectively on 145 and 17 patients reported a prevalence of 1:66.000 and 1:105,263 [12,13].

Since C1-INH-HAE is a genetic disease, the deficiency of C1-INH is present from birth. Nevertheless a minority of patients have perinatal angioedema symptoms. Patients typically begin to present clinical manifestations in childhood and attacks frequency often increase around puberty. Before the second decade of life the majority of patients manifest symptoms of angioedema [14]. Due to the rarity of the disease and to the fact that the clinical symptoms overlap with those of other forms of angioedema, C1-INH-HAE is frequently misdiagnosed. Consequently C1-INH-HAE patients may experience considerable delay between first symptoms and diagnosis. In a recent international observational study analyzing data of patients eligible for Icatibant treatment (Icatibant Outcome Survey, IOS) conducted in 8 European countries the mean delay in diagnosis of C1-INH-HAE patients was 12.8 years [15]. Previous nationwide surveys in France, Spain, and Denmark have reported mean delays in diagnosis of 12, 13.1 and 16.3 years, respectively [9,10,16].

In 1973 Agostoni et al. reported the first Italian family with C1-INH-HAE [17]. After that few centers in Italy have been active in diagnosing and treating this disease [14,18]. In 2007 a multidisciplinary panel of Italian experts in C1-INH-HAE met for a Consensus Conference held in Torino to define Italian guidelines for the diagnosis and therapy of C1-INH-HAE [19]. More recently, in 2012 an Italian network for C1-INH-HAE (ITACA) was established. ITACA, working together with the Italian Hereditary Angioedema Patients’ Association, collected data from C1-INH-HAE patients referring to 17 centers active in Italy. Here we provide the results of the analysis of these aggregate data.

## Methods

### Patients

Patients diagnosed with C1-INH-HAE at one of the 17 Italian centres from 1973 to December 2013 were included in the study. Diagnosis of C1-INH-HAE was based on personal and/or family history of angioedema and on C1-INH functional or antigenic plasma levels ≤50% of normal. All patients gave informed consent to use their anonymized data.

### Italian population

Data on demografic characteristics of the Italian general population were collected from the Italian Institute for Statistic (December 2013), www.istat.it.

### Data collection

For each patient the following information were collected from medical records: date of birth, date of diagnosis, condition (dead or alive), plasma levels of C1-INH and C4 at diagnosis. Patients were not on prophylactic treatment when complement parameters were measured. Independent families were identified and the first diagnosed member of a family was considered as proband.

### Laboratory methods

C1-INH antigenic and C4 were quantified using radial immunodiffusion or nephelometry; C1-INH function was measured using a chromogenic or an immunoenzimatic assay. Results were normalized as percentage of normal value (functional C1-INH normal range 70-130%; antigenic C1-INH normal range 70-115%; antigenic C4 normal range 60-140%).

### Diagnosis of C1-INH-HAE Type I and II

Patients were diagnosed as C1-INH-HAE type I when functional and antigenic C1-INH were ≤ 50% of normal, and as type II when functional C1-INH was ≤50% and antigenic was >50% of normal.

### Statistical analysis

Descriptive analysis of data from the Italian C1-INH-HAE population was performed; results were reported as mean, median, 25^th^ and 75^th^ percentiles range for each parameter. Differences in decade from C1-INH-HAE population and Italian population were analyzed using Chi square test at the 95% significant level and p-value < 0.05.

## Results

**Figure 1:**
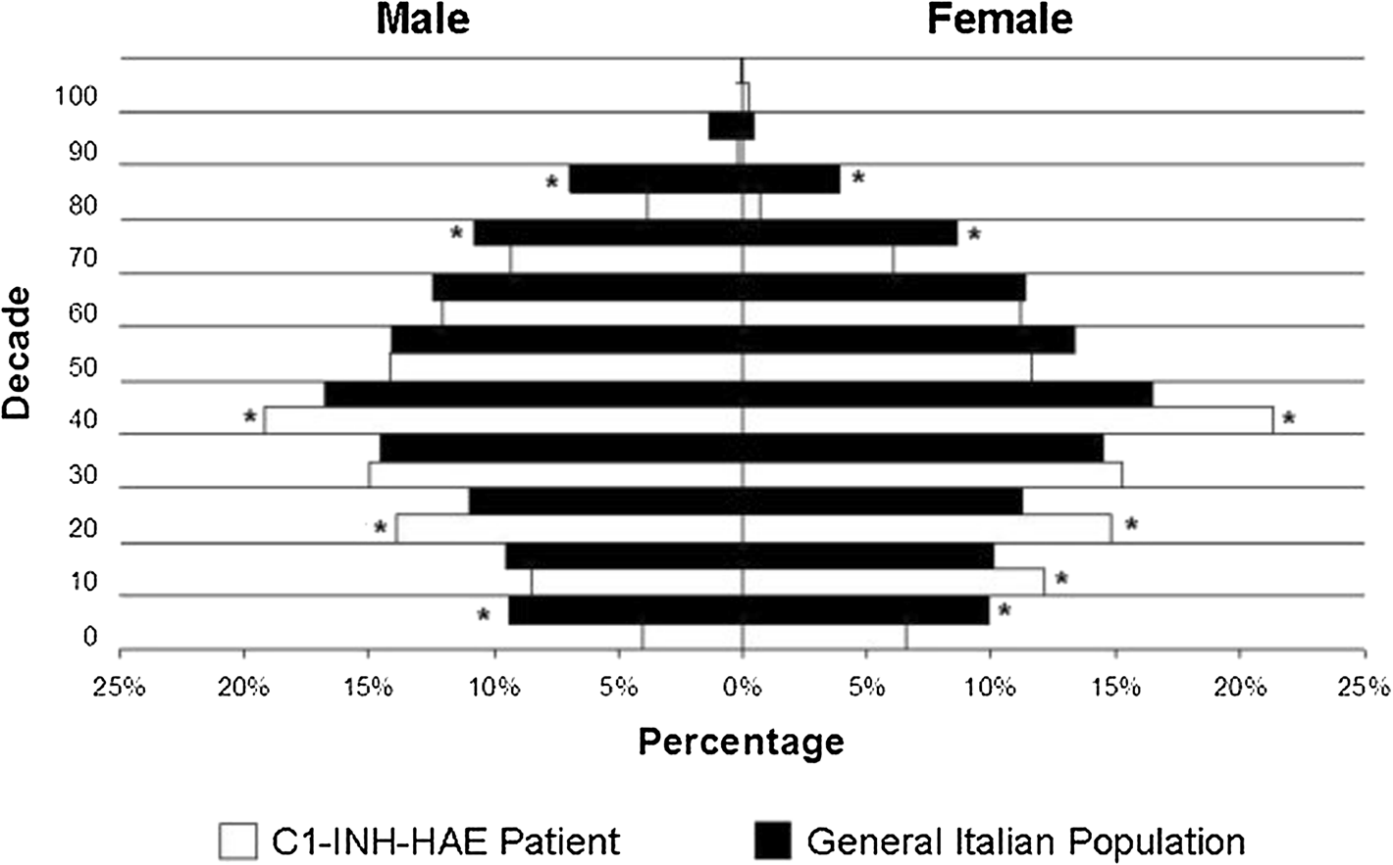
Distribution per decades of life of C1-INH-HAE patients compared to Italian population (*p < 0.05 versus general population).

Overall 983 patients (53% female) with C1-INH-HAE from 376 unrelated families were included in this analysis. Since 1973, 63 (6%) patients died; furthermore data from 3 patients were missing when analysis were performed. Accordingly, the minimal prevalence of C1-INH-HAE in Italy in 2013 is 1.54:100,000 inhabitants, equivalent to a prevalence of 1:64,935. Median age of patients is 45 (IQ 28-57), median age at diagnosis is 26 years (IQ 13-41). The majority of patients, 859 (87%), have C1-INH-HAE type I with median age at diagnosis of 25 (13-40); patients with type II are 124 (13%) with median age at diagnosis of 31 (IQ 16-49). Demographic characteristics and laboratory assessments are summarized in Table 1. Comparing the distribution per decades of life of patients and of the Italian population we found that patients are less represented in the first decade and after the 7^th^ decade compared to Italian population (p < 0.05) (Figure 1).

**Table 1:** **Demographic characteristics and laboratory assessments of Italian C1-INH-HAE patients**

	**Type I**	**Type II**	**Total**
Patients (%)	859 (87%)	124 (13%)	983
Gender (M/F)	407/452	55/69	462/521
Median age (years)	44	45	45
Median age at diagnosis (years)	25	31	26
Antigenic C1-INH, median value (%)	21 (IR 13-25)	96 (IR 64-150)	24 (IR 14-31)
Funcitional C1-INH, median value (%)	20 (IR 10-30)	19 (IR 10-30)	20 (IR 10-30)
Antigenic C4, median value (%)	20 (IR 10-25)	21(IR 12-30)	20 (IR 11-26)

**Figure 2:**
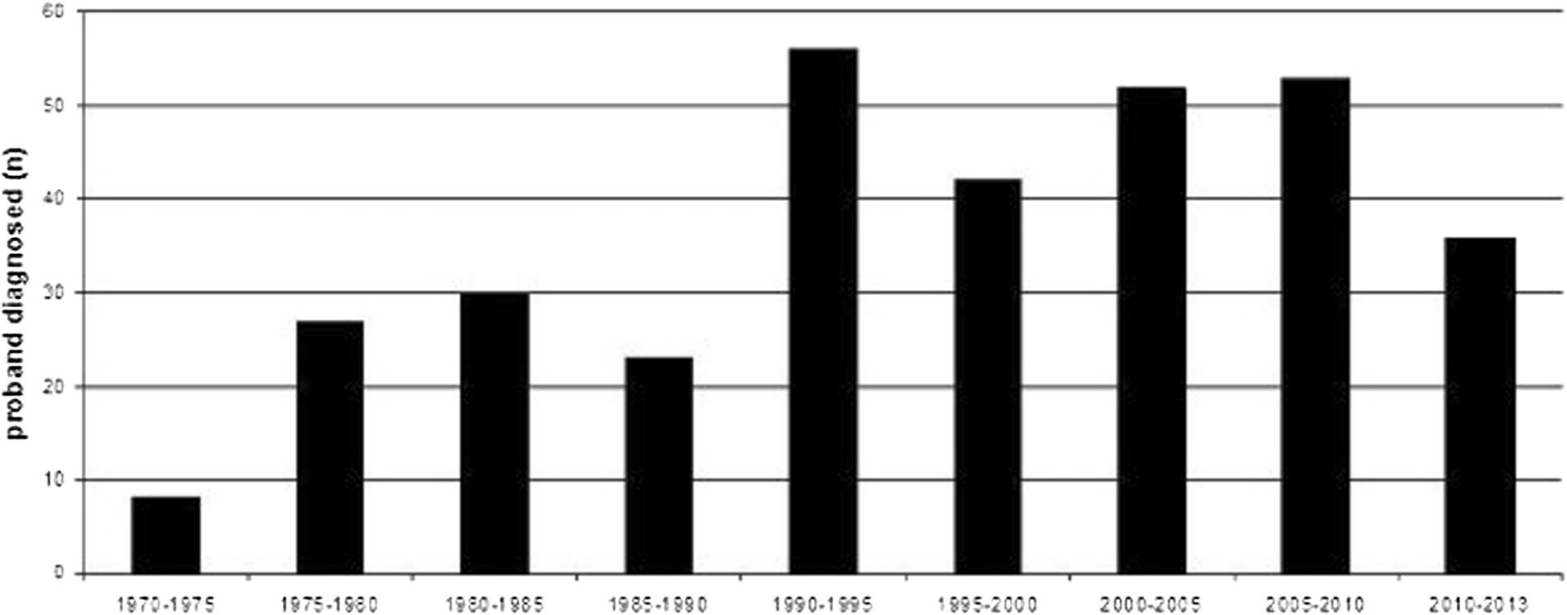
**Median age at diagnosis of probands from 1973 to 2013.** Numbers reported on top of histogram indicate new families diagnosed in each 5-year-period.

Of the 367 unrelated families, 338 (92%) have type I and 53 type II. “Families” with only one subject diagnosed as C1-INH-HAE are 157 (43%). The median age of probands at diagnosis is 34 years. Analyzing 5 year periods, starting from the first diagnosis in the seventies, the number of new probands increased until 1995 and remained stable afterwards (Figure 2). Functional C1-INH is ≤50% in 99% of patients. Antigen C1INH is ≤50% in 99% of type 1 patients. C4 antigen is ≤50% of normal in 96% of patients.

## Discussion

This first nationwide survey of C1-INH-HAE in Italy provides a minimum prevalence of 1: 64,935, the highest value ever registered for this disease compared to previous studies. Assuming that our centres collected data on the majority of Italian C1-INH-HAE population, patients not referring to these centres or not diagnosed certainly exist. It is therefore very likely that the real prevalence of C1-INH-HAE is higher approaching the prevalence of 1:50,000 usually reported in literature [20].

Male and female patients are equally represented as expected from an autosomal dominant pattern of inheritance of C1-INH deficiency.

Comparing C1-INH-HAE subjects and general Italian population for distribution in different decades of life, patients are less represented in the early and late decades. In the literature there is no evidence regarding life expectancy in C1-INH-HAE patients. The reduced number in the first and second decade for men can be explained with the delay in diagnosis, which is often made after the second decade of life [1]. The reduced number of patients in first decade can also be explained by the absence of symptoms and by not having systematically studied all the members of the family of every newly diagnosed C1-INH-HAE patient in the past. Interestingly, from 7th decades there are fewer individuals in patients’ group compared to general population. In 1992 Cicardi and Agostoni, analyzing the family histories of an Italian cohort of patients with C1-INH-HAE, reported that up to 50% of patients could have died for asphyxia [12]. This could explain the reason why in Italy C1-INH-HAE patients have had fewer chances, compared to the general population, to reach the late decades. We expect that diagnostic tools and therapeutic options available today will eliminate asphyxia as a common cause of death for C1-INH-HAE patients. This should lead to have patients and general population equally represented in late decades, unless other factors may affect life expectancy. The fact that C1-INH-HAE patients are less represented than general population in the late decades could been related to underdiagnosis in the past. The awareness of C1-INH-HAE has increased in recent years and it is possible that younger patients had more opportunities to be diagnosed than elder patients.

Median age at diagnosis in our patients is 26 year old, which is slightly later than in patients enrolled in IOS study (24.3 year old) [14]. Our study does not report data on delay in diagnosis (defined as time between onset of symptom and diagnosis), but assuming that the large majority of patients becomes symptomatic around puberty we could estimate a minimum delay in diagnosis of 10 years. This result is similar to the delay in diagnosis reported in previous studies. In the cohort of patients analyzed in IOS study the mean delay in diagnosis was 12.8 years [15]. In previous nationwide surveys in France, Spain and Denmark mean delay in diagnosis was 12, 13.1 and 16.3 years, respectively [9,10,16]. Furthermore, we found a considerable difference in the median age at diagnosis between patients with C1-INH-HAE type I and II, 26 and 31 years respectively. This difference was even higher in IOS study, 27.3 and 39.4 respectively. Since diagnosis of C1-INH-HAE type II is made measuring C1-INH function, the limited availability of this test may explain why diagnosing C1-INH-HAE type II is more difficult.

The number of new diagnosis, calculated as number of probands to avoid the effect caused by large family groups, increased up to 1996, likely due to an improving awareness for this disease. Since that time the annual rate of new diagnosis has remained stable without a trend in reduction suggesting that we are approaching the point at which most Italian C-INH-HAE patients have been diagnosed.

“Families” with only one subject diagnosed as C1-INH-HAE are 157 (43%). The number of *de novo* mutants reported in literature is approximately 25% of the families [3]. The high percentage of families with only one subject affected likely includes *de novo* mutants and patients with non-diagnosed family members. It is mandatory after diagnosis a new patients to evaluate other family members.

According to diagnostic criteria all patients had either C1-INH antigen or function ≤50%. In absence of published criteria to distinguish type I and II, we considered as type II patients with C1-INH antigen >50% of normal. According to these criteria, 2 patients with C1-INH antigen levels slightly above 50% should have been diagnosed as type II, but they were classified as type I because of, belonging to families whose other members were type I. Similarly, we should have considered as type I 12 patients with C1-INH antigen levels equal or slightly below 50% of normal despite other family members were type II. Finally, 6 patients, with personal and family history of angioedema and C1-INH antigen clearly below 50% of normal, had C1-INH functional levels slightly above 50%. These rare and small discrepancies between suggested diagnostic criteria and actual data highlight the need for comprehensive and thoughtful evaluation of clinical and laboratory findings before placing definitive diagnosis.

The actual value of antigenic C4 in diagnosing C1-INH-HAE has remained vague. Rosen et al in 1971 first proved the importance of C4 consumption for detecting C1-INH deficiency [21]. Our study demonstrates that C4 plasma levels >50% were rare in C1-INH-HAE patients and can exclude diagnosis of C1-INH-HAE with an error probability lower than 0.05.

## Conclusion

This nationwide survey on a large number of patients provided evidence that the estimated prevalence of 1:50,000 for C1-INH-HAE is probably close to the real prevalence in general population. Patients with HAE-C1-INH might have a shorter life expectancy compared to general population. An increased awareness of the disease, pedigree’s study, appropriate diagnosis and treatment is needed to reduce the discrepancy in the distribution of decade of life between general population and HAE-C1-INH patients. Measurement of C4 antigen, a cheap assay commonly available in laboratories, can exclude diagnosis of C1-INH-HAE with an accuracy > 95%. This parameter along with clinical data should be therefore considered for initial screening in differential diagnosis of angioedema.
